# Serologic Evidence of Lyssavirus Infection in Bats, Cambodia

**DOI:** 10.3201/eid1012.040459

**Published:** 2004-12

**Authors:** Jean-Marc Reynes, Sophie Molia, Laurent Audry, Sotheara Hout, Sopheak Ngin, Joe Walston, Hervé Bourhy

**Affiliations:** * Institut Pasteur du Cambodge, Phnom Penh, Cambodia;; † Institut Pasteur, Paris, France;; ‡ Wildlife Conservation Society, Phnom Penh, Cambodia

**Keywords:** Cambodia, Lyssavirus, Chiroptera, dispatch, bats

## Abstract

In Cambodia, 1,303 bats of 16 species were tested for lyssavirus. No lyssavirus nucleocapsid was detected in 1,283 brains tested by immunofluorescence assay. Antibodies against lyssaviruses were detected by enzyme-linked immunosorbent assay in 144 (14.7%) of 981 serum samples. Thirty of 187 serum samples contained neutralizing antibodies against different lyssaviruses.

The genus *Lyssavirus* belongs to the family *Rhabdoviridae* and includes seven species and one tentative species (*1*). Six of these seven species (or genotypes) have been isolated from bats. Rabies virus (RABV), responsible for most human rabies cases in the world, is associated with bats only in the Americas, and this association is currently responsible for most human rabies cases in North America. Lagos bat virus (LBV) and Duvenhage virus (DUVV) are found in Africa. European bat lyssavirus-1 (EBLV-1) and European bat lyssavirus-2 (EBLV-2) have been isolated in Europe. Australian bat lyssavirus (ABLV) has been detected in Australia. DUVV, EBLV-1, EBLV-2, and ABLV have been responsible for several fatal cases in humans (*2**–**4*).

In Asia, rabies infection of bats has rarely been reported. A human case of rabies with history of bat bite was first reported in 1954 in southern India. Two large surveys in the Philippines and in Malaysia failed to detect any rabid bats. Lyssavirus infection was detected in Thailand in a frugivorous bat, *Cynopterus brachyotis*, and in India in a frugivorous bat, *Pteropus poliocephalus* (*2*). Recently, new lyssaviruses (Aravan, Khujand, Irkut, and West Caucasian bat viruses) were isolated in southern Kyrghyzstan, northern Tajikistan, eastern Siberia, and the Caucasus from *Myotis blythi*, *M. mystacinus*, *Murina leucogaster*, and *Miniopterus schreibersi*, respectively (*5*). Furthermore, neutralizing antibodies against ABLV were detected in the Philippines in two frugivorous species and four insectivorous species, notably *M. schreibersi* (*6*).

In Cambodia, rabies is endemic, transmitted mainly by dogs. Since 1997, the Institut Pasteur du Cambodge (IPC) has received heads of suspected rabid domestic dogs from 11 of the 23 Cambodian provinces. Dogs from nine provinces had laboratory-confirmed rabies infection. Since mid-1995, ≈9,000 people per year have received free postexposure rabies treatment at IPC. No case of a human with rabies and a history of bat bite has ever been reported to IPC (*7*), but potential exposure to rabies from bats is often underappreciated (*8*).

Surveillance for lyssaviruses in bats in Southeast Asia has been very limited to date. No isolate has been identified, and no particular bat species has been implicated as a potential reservoir. We therefore conducted a survey to look for lyssavirus infection among bat populations in Cambodia.

## The Study

A total of 1,303 bats were sampled from 35 locations in nine Cambodian provinces (Figure). Of these, 467 came from restaurants in Phnom Penh and belonged to the species *P. lylei*. The other 836 animals were captured in nine provinces and belonged to 16 species representing six of the seven bat families known in Cambodia (Table 1, Figure). Bats were captured at roosts by hand and with hand nets, or along flyways by night with mist nets or hard traps. Anesthetized captured animals were euthanized by cardiac blood puncture, and their organs were collected. Sampling bats from restaurants was restricted to collecting blood and brain. All bats specimens were stored in 70% ethanol until species was identified.

**Figure:**
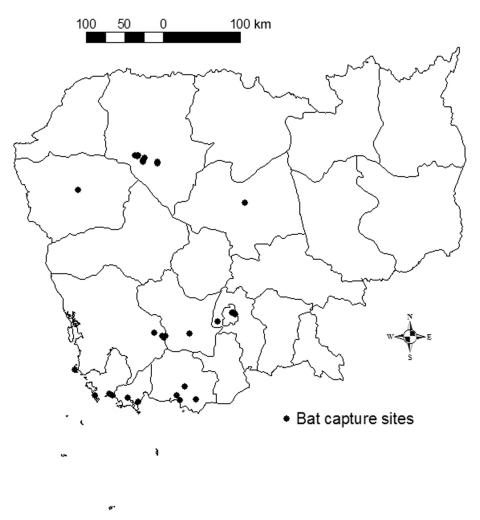
Location of bat capture sites during a survey on lyssavirus infection in bats in Cambodia, September 2000–May 2001.

**Table 1:** Lyssavirus enzyme-linked immunosorbent assay–reactive serum samples from bats, according to place of capture, Cambodia, September 2000–May 2001

Species (no. captured)	No. positive/no. tested (%)										
	Phnom Penh restaurants	Phnom Penh National Museum	Phnom Penh and vicinity	Siem Reap	Kirirom National Park	Kampot	Kompong Thom	Battam-bang	Kompong Som	Islands	Total (%)
**Frugivorous**											
*Cynopterus brachyotis* (1)					0/1						0/1 (0)
*Cynopterus sphinx* (83)			1/17	4/40	1/13					0/11	6/81 (7)
*Macroglossus sobrinus* (1)										0/1	0/1 (0)
*Pteropus lylei* (471)	25/224			0/4							25/228 (11)
*Roussetus leschenaulti* (16)					1/16						1/16 (6)
**Insectivorous**											
*Hipposideros armiger* (1)						0/1					0/1 (0)
*H. larvatus* (96)					0/16	2/40			1/36		3/92 (3)
*H. pomona* (6)									0/3		0/3 (0)
*Murina cyclotis* (1)					0/1						0/1 (0)
*Rhinolophus acuminatus* (2)					0/1				0/1		0/2 (0)
*R. luctus* (1)					0/1						0/1 (0)
*R. malayanus* (2)											
*Scotophilus kuhlii* (153)				21/110							21/110 (19)
*Tadarida plicata* (227)		26/104				23/78		8/33			57/215 (27)
*Taphozous melanopogon* (85)		0/6		1/1		1/27	3/6			1/32	6/72 (8)
*T. theobaldi* (157)		13/94				12/63					25/157 (16)
Total (1303)	25/224	39/204	1/17	26/155	2/49	38/209	3/6	8/33	1/40	1/44	144/981 (15)

Direct immunofluorescence assay (IFA) was performed on the brain of 1,283 (20 were not testable) bats to detect lyssavirus nucleocapsid (*9*). Rabbit antirabies nucleocapsid immunoglobulin G (Bio-Rad, Marnes-la-Coquette, France) was used at a concentration (2x) that reliably detects infection with the seven lyssavirus genotypes. None of the brains tested was positive. Attempts to isolate virus in newborn mice (*9*) from the brains of 24 bats that gave uncertain IFA results were unsuccessful.

Serum samples of bats were first screened for antibodies against lyssavirus by enzyme-linked immunosorbent assay (ELISA). Antigens were obtained from inactivated and titrated supernatants of BHK21 clone BSR cell cultures infected independently by four different genotypes circulating in bats, RABV (CVS strain), LBV, ABLV, and EBLV-1. These strains were chosen according to their ability to detect cross-neutralizing antibodies (*10*). Each supernatant was diluted to a titer of 6 x 10^4^ focus-forming units (FFU)/mL in carbonate buffer. Polysorp 96-well plates (Nunc, Roskilde, Denmark) were coated with 100 μL of RABV (CVS)/LBV mixture and 100 μL of ABLV/EBLV-1 mixture and incubated at 4°C overnight. The samples were diluted 1:50. Peroxidase-labeled protein A/G (Pierce, Rockford, IL) was used as conjugate. Three negative control serum samples and one positive control sample (equine rabies immunoglobulin 200 IU/mL) diluted 1:50 were included in each plate. Washing and diluent buffers, incubation, cutoff value, and positive definitions followed Rossi and Ksiazeck's technique (*11*). Of the 981 bat serum samples tested by this ELISA, 144 (14.7%) had a positive result (Table 1). Animals with ELISA-positive samples belonged to eight different species of both frugivorous and insectivorous bats.

ELISA test results were confirmed by using lyssavirus rapid fluorescent focus inhibition test (RFFIT) on 144 ELISA-positive and 43 ELISA-negative samples chosen at random. Each sample was tested independently against four different lyssaviruses: RABV, EBLV-1, ABLV, and LBV by an adaptation of RRFIT (*12*) and was considered positive for an average titer >42 after two independent assays.

Of the 146 samples with interpretable RFFIT results (76% were ELISA-positive), 10 (31%) of 32 samples from frugivorous bats and 20 (18%) of 114 samples from insectivorous bats were positive for neutralizing antibodies against at least one of the four genotypes (Table 2). In some cases when high titers against one virus were recorded, cross-neutralization occurred with other viruses. Geometric means for the 30 positive responses were 76.2 (n = 6), 71.9 (n = 13), 97.4 (n = 12), and 83.8 (n = 7) against CVS, EBLV-1, ABLV, and LBV, respectively. Eleven samples exhibited titers >100. Among the 10 RFFIT-positive frugivorous bats, neutralizing antibodies against ABLV were the most frequent (50%). Conversely, neutralizing antibodies against EBLV-1 were the most frequent (50%) among the 20 RFFIT-positive insectivorous bats. Positive responses were in the majority (83.3%) against these two viruses. The 30 bats with neutralizing antibodies belonged to frugivorous species, *Cynopterus sphinx* (n = 3) and *P. lylei* (n = 7), or insectivorous species, *Hipposideros larvatus* (n = 2), *Scotophilus kuhlii* (n = 5), *Taphozous theobaldi* (n = 2), *T. melanopogon* (n = 1), and *Tadarida plicata* (n = 10). No meaningful geographic trends were identified.

**Table 2:** Reactivity of serum samples from bats against four lyssaviruses with rapid fluorescent focus inhibition test, Cambodia, September 2000–May 2001

Virus^a^	Frugivorous bats (n = 32)		Insectivorous bats (n = 114)		All bats (N = 146)	
	Negative	Positive	Negative	Positive	Negative	Positive
RABV	30	2	110	4	140	6
EBLV-1	29	3	104	10	133	13
ABLV	27	5	107	7	134	12
LBV	28	4	111	3	139	7
Total^b^	22	10	94	20	116	30

^a^RABV, rabies virus; EBLV-1, European bat lyssavirus-1; ABLV, Australian bat lyssavirus; LBV, Lagos bat virus. ^b^Columns may add up to numbers higher than those mentioned in the total because of the reactivity of individual serum samples against more than one lyssavirus.

## Conclusions

This study reports the first evidence of anti-lyssavirus neutralizing antibodies in serum samples from insectivorous and frugivorous bats in Cambodia. These serologic data support the likely occurrence of rabies, possibly from a previously undescribed lyssavirus, among bats in Cambodia.

A simple ELISA was developed to detect antibodies against lyssavirus in bat serum samples as a first screening. The sensitivity and specificity of this test can be estimated by comparing its results with those of the RFFIT, which is considered the most effective and reliable method of detecting anti-lyssavirus antibodies. This comparison gives us a relatively high sensitivity (83%, n = 30) and a low specificity (27%, n = 116); therefore, ELISA could be used to test large numbers of samples. RFFIT, a time-consuming technique, could be used to double-check ELISA-positive samples. However, prevalence results obtained with ELISA should be considered cautiously because RFFIT was performed on samples selected according to ELISA results (and not performed simultaneously with ELISA on randomly chosen samples).

The threshold for RFFIT positivity chosen in this study was slightly higher than that used in recent bat studies performed in Europe (*3*) and the Philippines (*6*). Although no accepted standard for bat sera exists, the titer of 42 obtained in RFFIT against CVS-11 corresponds in our hands to a titer of 0.8 IU/mL using the World Health Organization (WHO) human standard. The arbitrary cutoff chosen during this study is then slightly higher than the arbitrary value (0.5 IU/mL) established by WHO as evidence of neutralizing antibodies against rabies having been induced after vaccination (*13*). This cutoff was chosen to avoid problems of test specificity because of hemolysis present in some specimens. The samples considered to be positive in this study should then be considered as highly indicative of anti-lyssavirus–specific antibodies.

None of the brain samples showed evidence of lyssavirus antigen or infectious particles. Similar studies did not succeed in detecting lyssavirus antigen or RNA in bats (*3**,**6**,**14**,**15*). Because the 1,303 bats collected in Cambodia during this study were healthy and belonged to 16 different species, the expected number of positive reactions would not be very high. One positive bat among them would indicate a global prevalence of active infection of 8 per 10^4^ bats, which would be high for randomly selected healthy bats.

Further investigation is needed to determine whether the circulation of lyssavirus in the Cambodian bat population poses a threat to human health. In the meantime, postexposure treatment should be considered in the event of a bat bite. The public, especially persons in close contact with bats (guano collectors, sugar palm tree collectors, persons with bats roosting in their houses), should be educated about the risk for rabies transmission from bats and should be encouraged to participate in surveillance by shipping specimens from sick bats for laboratory diagnosis of rabies.
